# Different attitudes during breastfeeding consultations when infant formula was given: a phenomenographic approach

**DOI:** 10.1186/1746-4358-6-1

**Published:** 2011-01-31

**Authors:** Sofia Zwedberg, Lars Naeslund

**Affiliations:** 1 Department of Woman and Child Health, Division of Reproductive and Perinatal Health Care, Karolinska Institutet, Stockholm, Sweden; 2 Department of Obstetrics, Karolinska University Hospital Solna, Stockholm, Sweden; 3 Department of Didactics, Stockholm University, Sweden

## Abstract

**Background:**

WHO and UNICEF believe that both antenatal and maternity care organizations are in an excellent position to protect and, if necessary, reinstate a culture that promotes breastfeeding, and that they are responsible for doing so. In Sweden, the number of breastfeeding women has been decreasing annually since 1996. Thus the aim of this study is to identify, describe and analyze the attitude midwives have towards the mother, child and breastfeeding when infant formula is given.

**Methods:**

From the theoretical standpoint of Buber's I-Thou and I-It concept, the different attitudes during breastfeeding consultations are interpreted. By using a phenomenographic approach based on 101 accounts of varying lengths from 39 midwives, different attitudes or approaches were identified.

**Results:**

Four different approaches are distinguished in the breastfeeding consultation. The first is the family as a whole, the second is mother and child as separate and equal, the third views the mother as superior and the fourth views the child as superior.

**Conclusions:**

The approach of the midwife is related to how she defines the overall perspective of the mother-child relationship and how she looks upon her relationship to the mother-child dyad. Her approach varies depending on whether she meets the mother and child as a subject, similar to herself, or whether she sees one of them as an object. A midwife may also take an outside position, as an object, thus excluding a genuine relationship with the mother. The results also indicate that health care professionals focus on parts of the whole instead of maintaining a holistic perspective.

## Background

Breastfeeding has both a biological and emotional impact on the health of the mother and the child. The close physical contact with the baby and the particular manner in which the child is breastfed are important elements in terms of bonding between mother and child and secure attachment [1,2]. This connection, or bonding, begins at birth, and increases the child's chances of continuing to receive its mother's care.

From the health perspective, breastfeeding is an important element of human well-being for both mothers [3] and infants[4].

WHO and UNICEF believe that antenatal and maternity care organizations are in an excellent position to safeguard and, if necessary, reinstate a culture that promotes breastfeeding, and that they are responsible for doing so. They also believe that health care staff members are in a good position to influence mothers who have recently given birth to begin breastfeeding. Breastfeeding is an indicator of good health, and care personnel are responsible for promoting behaviour that improves health. They must have the requisite knowledge that helps them to support, protect and promote breastfeeding [5].

Mothers can experience confusion and uncertainty in terms of how to act if staff members give them conflicting breastfeeding counselling [6]. However, there are numerous factors that influence whether mothers breastfeed [7,8]. Examples are socioeconomic status including for example age, education, civil status and social level as well as attitudes to breastfeeding and how people in the woman's surroundings look upon breastfeeding [8]. Health personnel knowledge in terms of breastfeeding is also significant [7]. Thus, it is important that personnel receive both the time and training needed to adhere to the ten steps of the WHO and UNICEF. There are also reports describing how fresh graduates feel pressure to do what more experienced members of staff say "they have always done", and practice does not always correspond to the Baby Friendly Hospital Initiative (BFHI) practices, even if the hospital has been evaluated and approved [9]. Midwives do not have the time to assist women who are experiencing breastfeeding difficulties because they are working under pressure and within poorly functioning organizations [10].

In Sweden, the proportion of women exclusively breastfeeding at one week, two, four and six months has been decreasing annually since 1996, both for one week, two, four and six months [11]. This raises the question of how midwives are acting in different breastfeeding situations. Thus the aim of this study is to identify, describe and analyze the attitude midwives have towards the mother, child and breastfeeding when infant formula is given.

## Method

An attitude can only be understood, of course, if there is awareness of how the person defines and perceives a specific situation. Defining and perceiving a situation is related to personal knowledge development [12,13]. Knowledge is personal and dependent on context; it develops in a dialectical interplay with the surroundings. Practical knowledge is most often associated with some sort of action. The knowledge itself can only be understood if there is understanding of how the individual looks upon and understands the action in a larger context [14]. Thus awareness in terms of action means that the actor has good reasons for his/her action as well as insight into a larger context of meaning for the action. For this reason, it is important to see beyond the action itself [14]. Doing so enables understanding of how the individual defined the context for the action, which then enables better understanding of its intention.

The phenomenographic research approach is a humanistic cultural approach that sees humans as creative individuals. Based on previous knowledge and experience, human beings perceive the phenomenon's meaning, structure and comprehensiveness in the life-world [14]. Perception or conception is an active, intentional action that enables human beings to create a context for that which s/he experiences [14,15]. How we understand our surroundings and our actions is the result of our collected experiences. Perceiving the external world also includes intentions and values [14]. Human beings have an innate desire to find solutions and structures in various situations, and previous experiences are utilized to do so; this can be said to relate to "real" thinking. Previous experiences and expectations affect how we perceive the life-world and how we act. Practical experience is important for learning how to perceive or conceive contexts of meaning [14,15] and for understanding comprehensiveness in health care. Professional knowledge is not only or even primarily a matter of what someone does, it is a matter of what one thinks about one's own actions [14] and how one perceives and defines situations [15]. This forms the background for understanding various attitudes.

### Design and selection

Data were collected in parallel with those from another study measuring how many children were given infant formula during their stay at maternity wards in Stockholm County during the spring of 1999. This had previously been reported by determination of how many children were given infant formula during their time at maternity wards [16]. This was repeated on seven different occasions. In conjunction with this survey, midwives (at one of the five participating hospitals) responsible for children who had received infant formula were asked to write a brief description (anonymously) in their own words of the events thus far, management of the events and the results of the actions taken. They were also asked to provide a personal reflection that focused on the positive or negative effects on the mother-child dyad of the infant formula and whether they believed that it would have been possible to take alternative action with equal or better results. The accounts and questionnaire replies for the first study were collected by an independent midwife. The accounts were given an ID number for each midwife to enable multiple accounts from a single person to be put together irrespective of the date of collection. The ID number code list was destroyed before the accounts were submitted to the researcher (SZ) to render the identification of individual midwives impossible.

One hundred and one accounts of varying lengths were obtained from 39 midwives. The researcher began by analyzing a small number of accounts and then added the others as work progressed. Initially, only accounts from midwives who had written three or four accounts were analyzed. This resulted in 29 accounts written by nine midwives which the researcher could then begin to analyze. The accounts were fairly evenly distributed over the various maternity wards. As work progressed, additional accounts were selected. The selection was based on all accounts, and the researcher added those conveying additional viewpoints and opinions.

### Analysis

Two aspects, *WHAT *and *HOW*, are distinguished in phenomenography. The content of a thought, i.e. what consciousness is focused upon, is called the *WHAT *aspect [17,18]. How one thinks about the content constitutes a process. The *HOW *aspect, or how process, helps distinguish the *WHAT *aspect. The *WHAT *and *HOW *aspects, thus, become mutually dependent upon one another. For example, one must have an idea of what learning is before it is possible to discuss how learning functions.

Normally phenomenography is based on interviews that provide empirical data and explore the HOW process due to a WHAT question. In this study, the first step involved identifying a WHAT aspect, i.e. a central theme or question that linked the written accounts. The researcher was able to finally identify the common denominator for the accounts, the WHAT aspect, as the "midwife's role for mother and child when breastfeeding does not completely function as it should".

The next step involved ascertaining the correlated HOW process. As support, the descriptions of the relationship between the midwife and the mother-child dyad were interpreted. The questions were: How did the midwives define the situation and how did they position themselves in relation to the mother-child dyad and to breastfeeding itself? How does the midwife regard the mother-child dyad, and what does she think about her own role? Is the mother-child dyad described as an object or subject according to Buber's definition [19]? The researcher studied how midwives position themselves in relation to mother-child and breastfeeding with help of Buber's definition. The "meeting" between the family or the mother is described here.

Buber says the world is twofold, that we have a world of experience and a world of relation. He describes the twofoldness as I-It and I-Thou. Buber writes that the fundamental words are word pairs rather than isolated words because they are inseparable. I-It represents a meeting between a subject and an object while I-Thou describes a meeting between two subjects. The meeting that offers admission to the world of relation can only take place with the person's entire being. I-Thou is communicated by genuine presence in a real relationship. Everything real in life is the I-Thou meeting. I become in relation to you. This is also how I get to know myself. In a true meeting, I simultaneously help form the Thou. A meeting is not possible unless I am capable of meeting the other person as an equal, i.e. as a subject like myself [19].

The researcher responded to the questions by taking notes and sketching out small images of how she interprets the relationship for each of the accounts. When the researcher had completed 35 compilations, she had a number of sketches that resembled one another. At that point she started to compare. The first step in the phenomenographic analysis was to read through all the excerpts to become familiar with the empirical material. The second step was to compile the responses to a certain question. The third step involved ascertaining the central aspects of the individual responses. It was then possible to begin classifying similar answers and, finally, to compare categories [20].

It was possible to distinguish four different approaches or attitudes in terms of defining the holistic perspective of the mother-child relationship and breastfeeding significant in terms of care measures. When comparing categories in phenomenographic research, the focus is on similarities and differences between the ways in which the phenomenon appears to the participants, i.e. identifying distinct ways of understanding or experiencing the phenomenon [21]. The last step involved establishing boundaries between the emerging categories. Thus, each of the small images contained criteria which made it possible to fit them into four separate emerging groups. The researcher then read the 101 accounts and found no reason to change her interpretation.

### Ethical considerations

All midwives participating in the study were informed both in writing and verbally of why they were being asked to anonymously record their reflections on mother-child dyads in which the child had been given infant formula, and that participation was entirely voluntary. The midwives were given assurance that no one other than themselves had access to the list containing the code key, i.e. tracing of authors of the accounts was impossible. They were also given assurance that their accounts would be carefully safeguarded to prevent them from being used in other contexts. Consent was given by writing down reflections and submitting them, together with other relevant forms, to the midwife responsible for collecting the information.

The head of the hospital clinical department gave approval for the study (4 January 1999). Ethics committee approval is not necessary for research about staff [22]. The hospital referred to common ethics guidelines and the study adhered to the guidelines for nursing research in the Nordic countries [23].

## Results

By using the 29 selected accounts, the researcher were able to identify a phenomenon as the common denominator, the *WHAT *aspect in accordance with the phenomenographic research tradition. The *WHAT *aspect was identified as the "midwife's role for mother and child when breastfeeding does not completely function as it should". *HOW *did midwives discuss the *WHAT *aspect? Based on this perspective, the researcher identified four attitudes. These approaches or attitudes were related to how the overall perspective adopted by midwives for the mother- child relationship and breastfeeding was defined. The attitudes also influence care measures. The four different groups are organized as follows: The family as a whole, mother and child separate and equal, mother viewed as superior and child viewed as superior.

### The family as a whole

**Figure 1:**
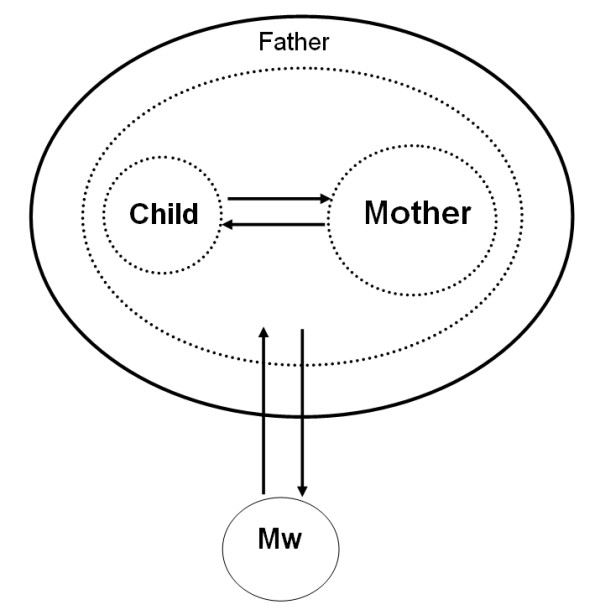
**The family as a whole**. This is a complex holistic view of the family in which the elements collaborate with one another. The mother-child relationship is an integrated unit, and breastfeeding has an aspect beyond food. It is characterized by a relation with two subjects, the midwife and the mother-child dyad (as a unit of a family).

The midwife sees her own role as providing support to the family. She sees the mother-child relationship as an integrated whole. Breastfeeding is seen as constituting an important emotional bond between mother and child to be encouraged and supported as much as possible. The midwife has both a medical and a psychological perspective. Care measures that support the whole are emphasized. Shaping a long-term breastfeeding strategy together with the mother is seen as an important step when breastfeeding does not go as planned. The midwife has a great deal of trust in the woman's ability and treats her as an equal subject with the result that the woman's belief in her own capability is reinforced. The holistic perspective is the most important factor. See Figure 1.

*The child was born in week 37. The child had lost > 10% of its body weight by the third day; according to hospital instructions, this condition requires supplementary feeding*.

*I look at the child's faeces and see that it has begun to be of the type that results from breast milk. I watch a breastfeeding session, and see that everything is as it should be. The child sucks and attaches well, and is well positioned at the breast*.

*The mother is informed that she has resources of her own and that it is important both to get plenty of rest and to eat and drink properly. She is also told that she needs the child and that the child has a great need of tactile, bodily contact with her. We shape a breastfeeding strategy together. The mother wills breastfeed every other hour and express milk by hand. She can use oxytocin spray*.

*The mother pumped and breastfed every other hour during the day so that she could rest at night. She gave the expressed milk by cup. We also made arrangements so that her husband could sleep at the hospital. The child had gained weight nicely by the following day*. (ID 193)

### Mother and child separated and equal

This describes a view of mother and child as equivalent, but not integrated. The parts are more important than the whole. The role of breastfeeding in terms of the mother-child relationship is unclear. However, it is characterized by a subject-object relationship where the midwife is the subject and the mother and her child are the objects.

**Figure 2:**
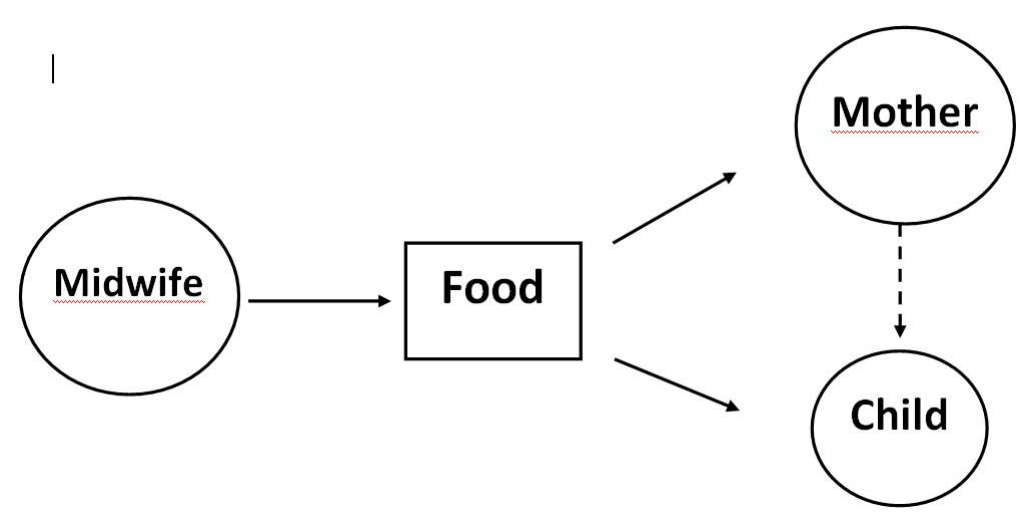
**Mother and child separated and equal**. This approach sees mother and child as equivalent, but not integrated. The parts are more important than the whole. The role of breastfeeding in terms of the mother-child relationship is unclear. However, it is characterized by a subject-object relationship in which the midwife is the subject and the mother and her child are the objects.

The midwife tries to help both the mother and child feel contented. Infant formula is given in the short-term in order to create peace and quiet for the mother and child. The importance of breastfeeding in terms of the mother-child relationship is unclear. The midwife often gives the impression of wishing to accommodate the mother. She solves the mother's problems *for *her by giving infant formula, which is why the subject-object relationship is selected, but only temporarily. A long-term plan for helping the mother to breastfeed successfully is not set up. This would have strengthened the bond between mother and child. See Figure 2.

*The child was crying. It had eaten a great deal but was not satisfied. The mother was tired and worried. I think the mother was quite satisfied that the child received infant formula. The mother's expressed milk was insufficient to calm the child*. (ID 260)

*I believe the mother is satisfied. She feels she can learn to breastfeed at home and knows where to turn to if she needs help when she gets home*.

*The positive aspect of giving infant formula in this case is that the child felt satisfied, which is what the parents desired*.

*They were able to sleep a little more and gain a little more energy*. (ID 192)

### The mother viewed as superior

In this attitude, the needs of the mother are viewed as superior to those of the child. The midwife functions reactively and primarily attempts to satisfy the needs of the mother, possibly at the expense of the child. Breastfeeding is primarily seen as a way to feed the child. This is characterized by an object-subject relationship, where the midwife is the object and the mother is the subject.

**Figure 3:**
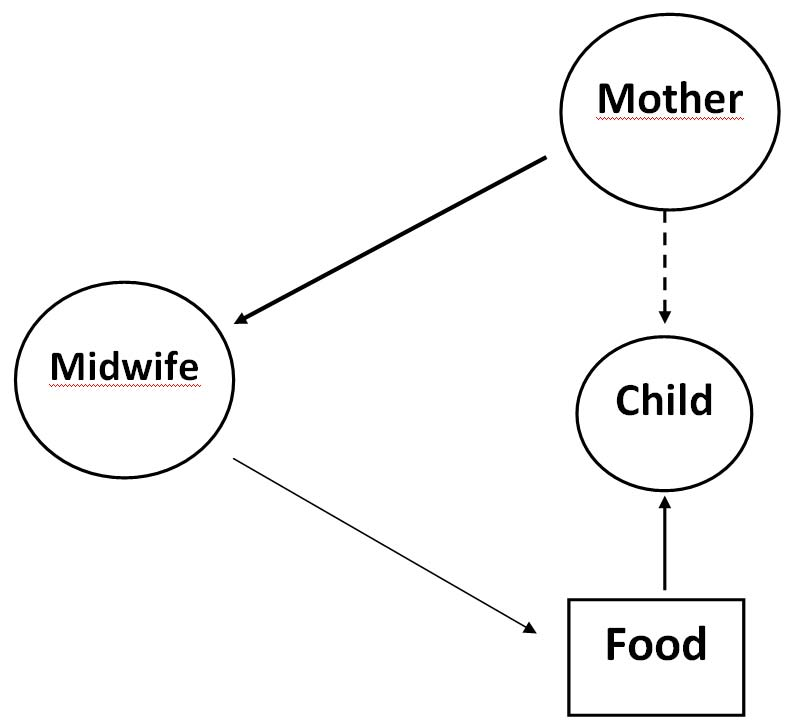
**The mother as superior.** The needs of the mother are superior to those of the child. The midwife functions reactively and primarily attempts to satisfy the needs of the mother, possibly at the expense of the child. Breastfeeding is primarily seen as a way to feed the child. Characterized by an object - subject relationship.

The midwife primarily sees her role as satisfying the needs of the mother. The mother-child relationship is not in focus; rather, concentration is on technically resolving "food concerns" based on the mother's wishes. The mother's wishes are considered to be more important than the child's. Infant formula is often given as a simple measure that temporarily saves time without first attempting to discern the mother's underlying wishes. The midwife often adopts the role of wanting to accommodate the woman without first attempting to influence her with her knowledge. She positions herself at the margin and the meeting is characterized by an object-subject relationship. See Figure 3.

*The child is large and hungry -- wants to eat frequently - but the mother feels she doesn't have the energy and has asked for infant formula. I am uncertain whether the mother thinks breastfeeding is important or not. Gave infant formula when the mother asked for it*. (ID 197)

*Completely acceptable to give infant formula since the mother had a C-section and finds pumping by hand difficult*. (ID 177)

### The child viewed as superior

The main concern of the midwife rests with the child, and she lacks confidence in the mother's ability. Breastfeeding offers nothing more than food and can easily be replaced. Characterized by a subject-object relationship, where the midwife is the subject and the mother and her child are the objects.

**Figure 4:**
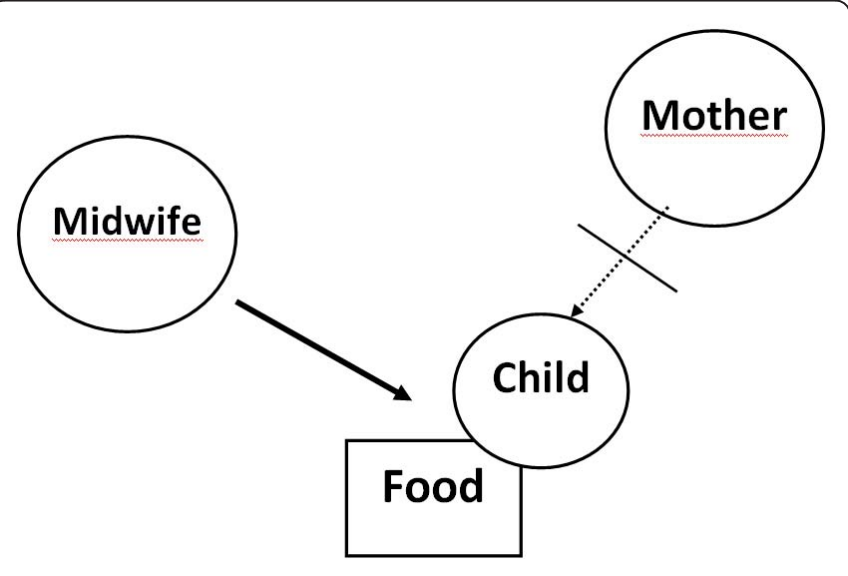
**The child as superior**. The midwife's main concern rests on the child, and she lacks confidence in the mother's ability. Breastfeeding offers nothing more than food and can easily be replaced. Characterized by a subject-object relationship in which the midwife is the subject and the mother and her child are the objects.

This group is characterized by short-term planning in which measures are often taken to reduce the midwife's own feelings of concern. The midwife focuses on the child and does not reflect upon the integrated mother-child unit in which the mother's own ability to care for the child may also have an emotional value. The midwife does not consider any care measures that may reinforce a good mother-child relationship. The midwife does not dare trust the mother, and the meeting is characterized by her belief that, as a health care professional, she knows best what the child needs. Problematic breastfeeding is of secondary concern. The issue of a mother who feels joy and reinforced self-confidence as a result of being able to satisfy her own child's nutritional needs is not touched upon. Breastfeeding is not viewed upon as something positive for the mother-child relationship; rather, it is solely seen as a way to feed the child. See Figure 4.

*According to the midwife on duty, the patient has an abundance of milk. The patient both expresses milk by hand in order to feed by cup and breastfeeds the child*.

*I believe the child should have received infant formula earlier. The child was born prematurely and has not gained weight as it should. The period of care will now be unnecessarily long. This is negative in light of the existing shortage of space*. (ID 182)

*It would have been possible to wait longer than age 1.5 days before giving the little boy infant formula since he sucked well on two occasions: right after delivery and at age 15 hours (no more infant formula needs to be given). If the midwife still feels concerned for the child and wants to give it food, I believe she should first really try to help the mother express her own milk. An electric pump can be utilized if the hand pump doesn't produce enough milk*. (ID 229)

## Discussion

The purpose of this research was to provide insight into midwives' different approaches or attitudes during breastfeeding consultations.

A new mother who experiences difficulty breastfeeding often feels vulnerable and exposed. She needs affirmation; not giving her affirmation can result in her experiencing even more stress and insecurity [24], thus increasing the distance to the child. Protecting and promoting breastfeeding should primarily involve seeing mother and child as an integrated unit, and, to the extent possible, attempting to bring the two together. When infant formula becomes necessary as a result of medical issues, it is even more important that the mother feels as though she is part of the process and important to her child. Breastfeeding is based on collaboration between mother and child, and the mother often requires the midwife's support in the process. Not feeling respected or supported by the midwife during delivery resulted in women seeking alternative care for their next delivery [25]. Central aspects for a positive experience involve the woman feeling that she takes part in decisions and in her having confidence in the midwife as a result of mutual confidence. Feeling involved does not always mean sharing responsibility for decisions taken. Rather, it involves feeling respected and listened to, and being familiar with the various perspectives even if the decision-making process is left to those who are responsible for medical issues [26]. This also emphasizes the importance of mutual confidence. The midwife's role, thus, should be to balance normalcy and the medical perspective. Genuine dialogue between the midwife and the new mother are necessary to achieve this balance. Being able to support, protect and promote breastfeeding [5] is based on an attitude in which the mother and child are seen as an integrated unit and in which they are important for one another. Berg outlined a care model for assisting mothers whose pregnancy is high risk based on a dignified, protective relationship to the mother and in which natural and medical perspectives are balanced as a result of well-integrated knowledge [26]. Genuine care can in this way be administered to the genuine woman [27]. The mother-child relationship could also be emphasized and safeguarded in the same manner. Mother and child are important for one another, and the strong emotional bond between them [2,24] should be reinforced in every manner possible; the bond is important to the health of both mother and child.

The normal way to collect data in phenomenographic studies is to carry out in-depth interviews to explore how individuals experience, perceive or conceive a phenomenon in different ways. The decision to have midwives record their reflections in writing was based, in part, on the importance of being able to ensure their anonymity. It was also carried out in a desire to disrupt day-to-day work as little as possible. Since the researcher is a midwife herself, she knows or knows of several of the midwives who recorded their reflections, and it was important that the researcher be unable to associate any of the accounts with a specific author at any point in the process. The midwives decided themselves whether they wished to record their opinions in writing or not, as well as how many accounts they wished to write if they were responsible for more than one child who was receiving infant formula at the time of the study. The accounts also varied a great deal in scope -- from a couple of lines up to two pages of text. All of the midwives agreed to share their viewpoints and opinions, and the 101 accounts correspond to the number of children receiving infant formula.

The accounts were often written very quickly and without a great deal of reflection or consideration. At first, the author considered this to be a major disadvantage since the ambition was to obtain a second -order perspective in terms of how the outside world was understood [13,18,21]. Interviews would probably have been a better method to get close to participants and to go in-depth. However, by utilizing the underlying philosophy that intimately links the *WHAT *and *HOW *processes and which designates humans as individuals who create meaning and who are intentionally focused [28], the researcher was able to recognize the value of speed and that choice of words can reveal underlying values [29]. In an interview, the respondent may take the time to reflect more. In the present case I captured the first thing that came to mind and therefore detected the "real thinking".

Some of the accounts pertain to the midwives themselves as caregivers while others pertain to care previously administered by other colleagues. Recovering the real phenomenographic question, the *WHAT *aspect, posed a challenge in this study.

The result indicates that mothers and children are looked upon as two separate individuals in the organization rather than as an important unit. In this way, care can be said to reinforce one-dimensional thinking rather than multi-dimensional holistic thinking.

Since a person's knowledge is developed in a dialectical interplay with his/her surroundings [12], surroundings probably also play a large role for both *WHAT *and *HOW *in terms of how one learns to perceive what is important. When humans create meaning between themselves and the outside world, a framework is formed on which knowledge is based. Learning takes place when humans acquire a new relationship to the outside world. After learning has taken place, knowledge is based on humans obtaining a slightly different perception of the outside world than they had before, consciously or unconsciously [18]. Defining and perceiving a situation is related to personal knowledge development [14]. The results do not indicate that individual midwives necessarily act as *one single *group; different approaches or attitudes exist. A holistic view in health care entails, for example, achieving an encounter between two subjects. It is a matter of a professional approach between the patient and the caregiver at the same time as it is a relationship between two individuals [30].

## Conclusion

The approach/attitude of the midwife is related to how she defines the holistic perspective of the mother-child relationship and how she looks upon her relationship with the mother-child dyad. Her approach is different depending on whether she meets the mother and child as a subject, similar to herself, or if she sees one of them as an object. A midwife may also position herself on the outside, as an object, excluding genuine relationship with the mother.

## Competing interests

The authors declare that they have no competing interests.

## Authors' contributions

SZ performed all the research steps of this study. LN is her supervisor and has supervised the analysis and the writing process. Both authors read, commented and approved the final manuscript.
